# Severe Pasteurella multocida Infection in a Neonate: A Case Report and Literature Review

**DOI:** 10.7759/cureus.70239

**Published:** 2024-09-26

**Authors:** Naohiko Maejima, Kensuke Shoji, Yoshiki Takezawa, Hiroyuki Aiba, Hiro Nakao, Mikiko Miyasaka, Chikara Ogimi, Shotaro Matsumoto, Satoshi Nakagawa

**Affiliations:** 1 Critical Care Medicine, National Center for Child Health and Development, Tokyo, JPN; 2 Infectious Diseases, National Center for Child Health and Development, Tokyo, JPN; 3 General Pediatrics and Interdisciplinary Medicine, National Center for Child Health and Development, Tokyo, JPN; 4 Radiology, National Center for Child Health and Development, Tokyo, JPN

**Keywords:** animal bite, antimicrobial therapy, neonates, pasteurella multocida, surgical intervention

## Abstract

*Pasteurella multocida* is a well-known cause of skin and soft tissue infections resulting from animal bites. The patient was a newborn male with no perinatal abnormalities except for a cephalohematoma on the left parietal region. At 10 days of age, he had a bite wound to the cephalohematoma from an indoor dog. The wound was very mild and there was no bleeding. At 12 days of age, he visited a local hospital with a fever and poor feeding. At presentation, he was in septic shock and was transferred to the intensive care unit of the hospital. On day three of hospitalization, *P. multocida* was identified in the blood culture. On day four of hospitalization, CT of the head revealed multiple low-density lesions in the cerebral parenchyma, and aspiration of the cephalohematoma was performed. A culture of the aspirated fluid also grew *P. multocida*. On day five of hospitalization, the patient underwent drainage of the cephalohematoma. The patient developed hydrocephalus and worsening cerebral edema injuries, ultimately resulting in severe neurological sequelae. We summarized previous reports on *P. multocida* infections in children 60 days old or younger. Of the reported cases, neonates accounted for a majority of cases. In addition, nontraumatic exposure was more common than traumatic exposure. The patients requiring surgical intervention and those with neurological sequelae were all neonates. Neonatal *P. multocida* infection can cause severe systemic illness and neurological sequelae, even in the absence of traumatic exposure.

## Introduction

Interest in animal bites has grown in recent years, especially with an increase in the number of pet owners. *Pasteurella multocida* is the most common organism that causes infectious diseases after a dog or cat bite [1]. It is a facultative anaerobic gram-negative bacillus known to be common in the oral cavity of animals, except humans, accounting for 70% to 90% of cat bites and 50% to 66% of dog bites [2].

Skin and soft tissue infections, including cellulitis and abscesses, are commonly caused by animal bites, with severe cases progressing to systemic infections, such as septicemia and bacterial meningitis [3,4]. Although several cases of *P. multocida* infection in young children have been reported [5,6], information on the clinical course and prognosis remains limited. Here, we report a case of severe neonatal *P. multocida* infection following a dog bite to the head. Previous cases of *P. multocida *infections in neonates and young infants were reviewed.

## Case presentation

**Figure 1:**
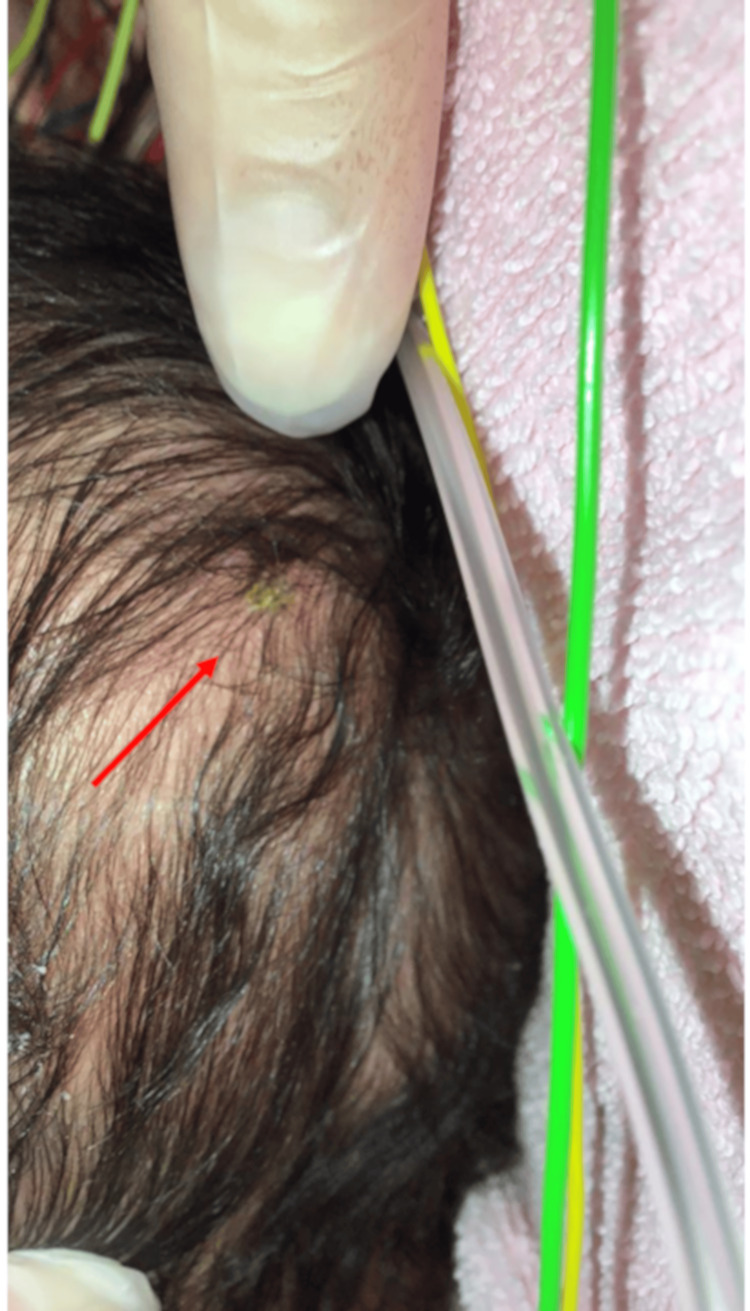
The cephalohematoma presented as a 10 cm x 8 cm-sized elastic and soft mass with slight crusting on the surface. No localized redness or heat, and no apparent puncture point was observed.

The patient was a 12-day-old male with an insignificant perinatal history, except for a cephalohematoma on the left parietal region from birth. Two days prior to hospitalization, an indoor dog caused a very minor bite to the cephalohematoma. As the wound did not bleed and no external findings were noted, it was disinfected by a primary care physician, and an antibiotic ointment was applied, but no systemic antibiotics were prescribed. The day before hospitalization, the patient developed a fever and poor feeding. At the initial presentation at a local hospital, fever, altered mental status, and circulatory failure were observed. Sepsis was suspected, and cefotaxime and an isotonic crystalloid solution (60 mL/kg) were administered. The patient was transferred to our hospital for intensive care. At the time of hospitalization, the patient presented with the following vital signs: body temperature 39.4 °C; heart rate 200/min; blood pressure 82/43 mmHg; saturation of peripheral oxygen (SpO2) 99 % (O2 6 L/min); Glasgow coma scale E3V4M4. A 10 cm x 8 cm-sized elastic soft cephalohematoma extending from the left parietal to the temporal region was noted. A very small crust with mild erythema was observed on the surface of the cephalohematoma (Figure 1).

Laboratory findings demonstrated a decreased white blood cell count of 1410 /μL (neutrophils 35.6%), an elevated C-reactive protein level of 15.4 mg/dL, and a lactate level of 7.6 mmol/L (Table 1).

**Table 1:** Laboratory findings WBC: White blood cell count; PT: Prothrombin time; PT-INR: Prothrombin time-international normalized ratio; APTT: Activated partial thromboplastin time; AST: Aspartate aminotransferase; ALT: Alanine aminotransferase; TP: Total protein; BUN: Blood urea nitrogen; Na: Sodium; K: Potassium; Cl: Chloride; CK: Creatine kinase; CRP: C-reactive protein

Variable	Value	Variable	Value
WBC	1410/µL	AST	37 U/L
Neutrophil count	35.6%	ALT	11 U/L
Lymphocyte count	51.7%	TP	4.4 g/dL
Hemoglobin	14.6g/dL	Albumin	2.9 g/dL
Hematocrit	45.5%	BUN	6.0 mg/dL
Platelet count	16.7×10^4^/µL	Creatinine	0.21 mg/dL
PT	17.7 second	Na	135 mEq/L
PT-INR	1.55	K	4.5 mEq/L
APTT	57.2 second	Cl	102 mEq/L
Fibrinogen	475 mg/dL	CK	124 U/L
D-dimer	2.5 µg/mL	CRP	15.38 mg/dL
Total bilirubin	8.01 mg/dL	Lactate	7.6 mmol/L
Direct bilirubin	1.19 mg/dL		

**Figure 2:**
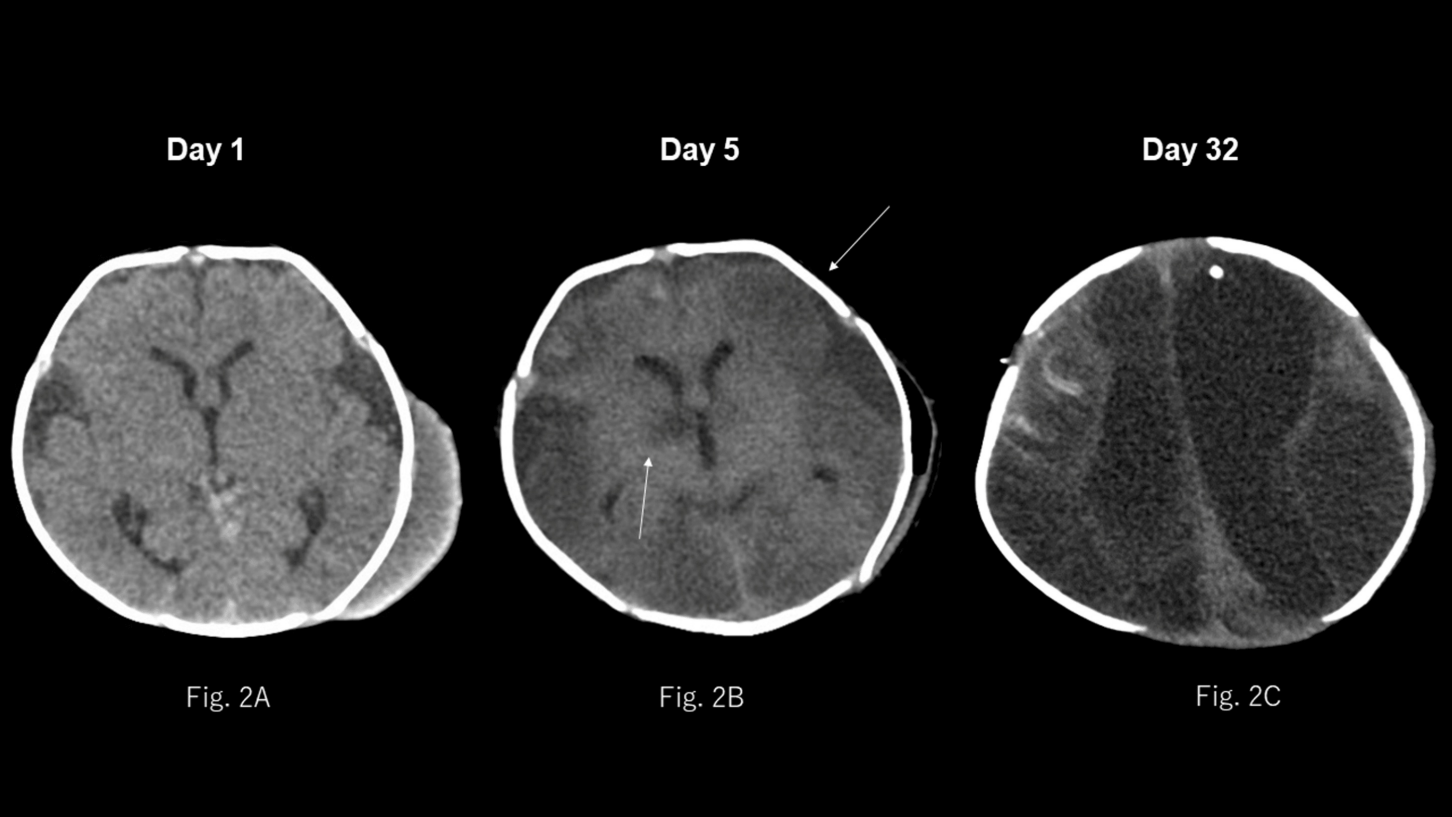
CT images of the head taken without contrast during treatment 2A: On day 1 the cephalohematoma is seen extending from the left temporal to the parietal region with no intracranial abnormalities. 2B: On day 5, the follow-up CT of the head after drainage revealed low-density lesions bilaterally in the cerebral hemispheres and basal ganglia. Subdural fluid collection was observed bilaterally in the frontotemporal regions. 2C: On day 32, laminar necrosis of the right frontal cortex was observed. The CT scan performed on day 32, upon discharge from the intensive care unit, showed bilateral enlargement of the lateral and third ventricles. Extensive low-density areas were observed in both cerebral hemispheres. Cystic encephalomalacia was observed.

An initial CT scan of the head revealed a known cephalohematoma but no evidence of abnormal findings, such as intracranial hemorrhage or abscess formation (Figure 2A).

**Figure 3:**
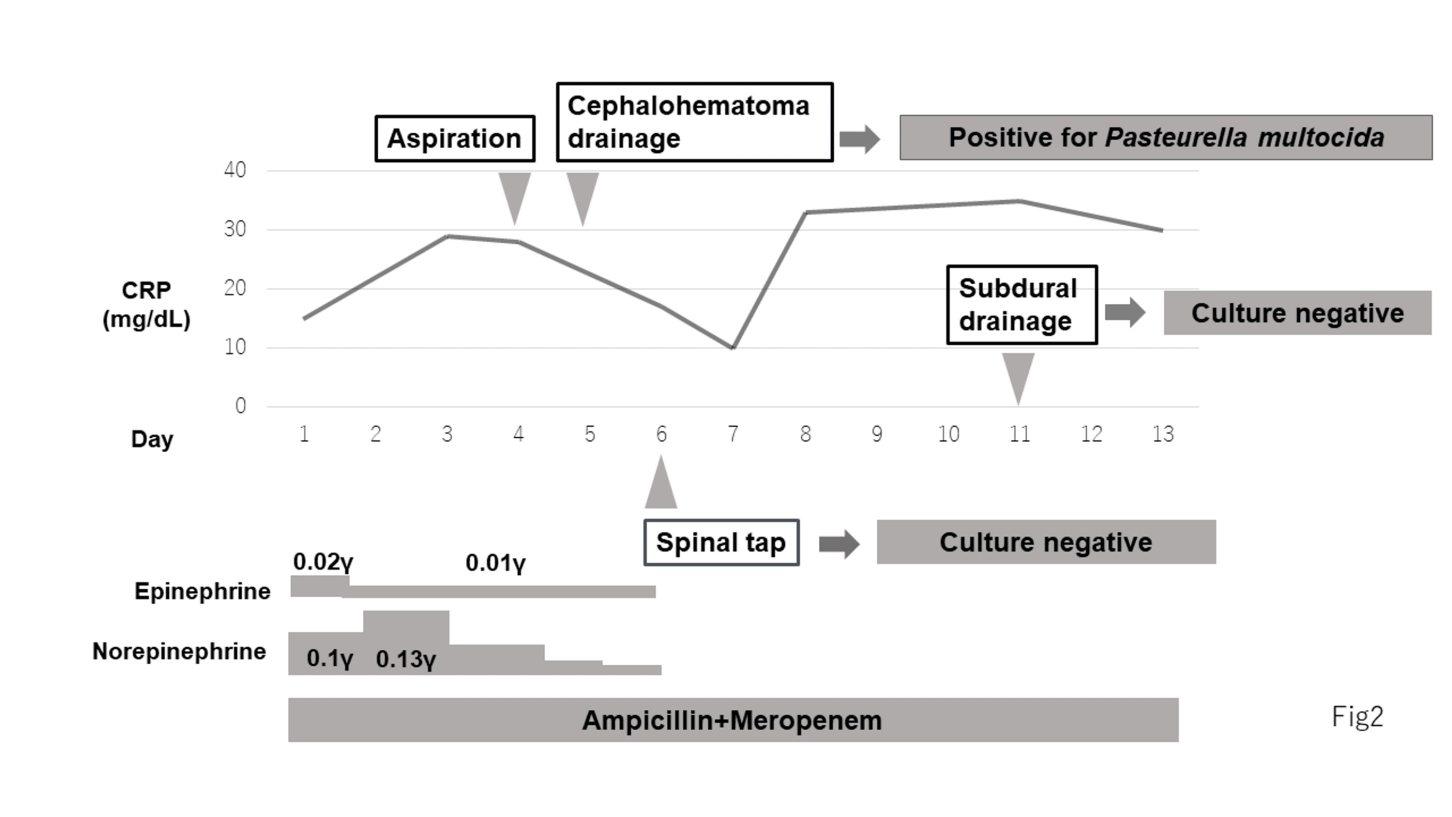
The clinical course of the patient *Pasteurella multocida *was detected in the puncture aspiration and drainage specimens on days four and five of hospitalization. Bacterial cultures from a lumbar puncture on day six and drainage specimens on day 11 were both negative. CRP: C-reactive protein

The patient was admitted to the pediatric intensive care unit (PICU) for further management of septic shock. Mechanical ventilation, antibiotics (meropenem 40 mg/kg every eight hours for gram-negative bacilli and anaerobe coverage and ampicillin 75 mg/kg every six hours for *Listeria monocytogenes *and *Streptococcus agalactiae* coverage), and inotropes were initiated. Figure 3 shows the clinical course of the patient after admission.

**Figure 4:**
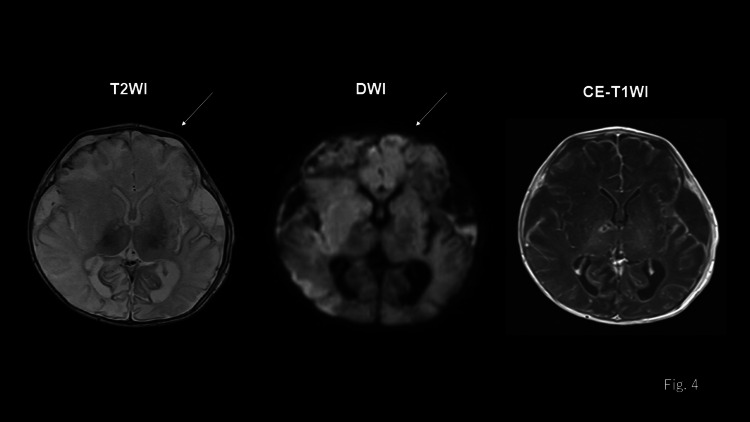
MRI of the brain on day 11 of hospitalization Bilateral subdural fluid collections in the frontotemporal regions revealed on a T2-weighted image.

Furthermore, the site of the bite displayed no evidence of infection, and no puncture or drainage of the cephalohematoma was performed at the time of admission. On day three of hospitalization, the blood culture was positive for *P. multocida*. As the patient’s condition did not improve, aspiration of the cephalohematoma was performed on day four of hospitalization, and gram-negative bacilli were identified in the aspirated specimen by gram staining. As infection of the cephalohematoma was strongly suspected, it was washed and drained on day five of hospitalization, and *P. multocida* was detected in the aspirated fluid. A follow-up CT scan of the head performed after drainage revealed multiple low-density lesions, indicating edematous or ischemic lesions (Figure 2B). After drainage, the patient became afebrile, and the inotropes were terminated. A cerebrospinal fluid examination performed on day six of hospitalization did not reveal any bacteria. An MRI of the brain was performed on day 11 of hospitalization. Subdural fluid collections suggestive of subdural empyema were observed bilaterally in the frontotemporal regions (Figure 4).

**Figure 5:**
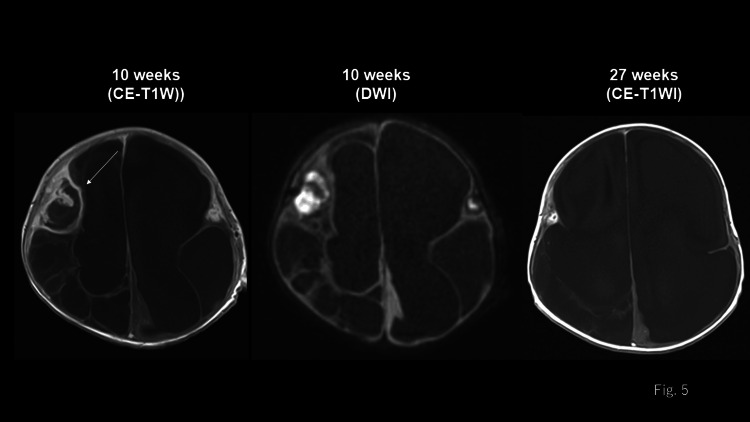
Serial contrast-enhanced-T1-weighted images of the brain Cystic parenchymal changes in the brain were noted on MRI 10 weeks after hospitalization. A diffusion-weighted image shows high signal intensity, suggestive of brain abscess bilaterally in the frontal lobes, and the contrast-enhanced T1-weighted images show ring enhancement (arrow). The MRI at 27 weeks revealed the progression of encephalomalacia. The brain abscess with ring enhancement is observed to be reduced in size.

Subdural drainage was performed on the same day. Thereafter, hydrocephalus developed, and cerebral ischemic changes worsened over time (Figure 2C). Cystic encephalomalacia subsequently developed. Additionally, respiratory dysregulation and central enuresis gradually developed. A tracheostomy was performed on day 18 of hospitalization and the patient was discharged from the PICU on day 32 of hospitalization. A follow-up brain MRI showed residual abscesses (Figure 5). Therefore, antimicrobial therapy was continued, which eventually ended after 28 weeks of hospitalization.

Six months from onset, the patient was mechanically ventilated, and his pediatric cerebral performance category scale score was expected to be equivalent to four, which is indicative of activities of daily living (ADL) milestones below the 10th percentile and excessive dependence on others for ADL [7].

## Discussion

We encountered a neonatal case of sepsis caused by *P. multocida* following a very mild dog bite, resulting in severe neurological sequelae. To characterize *P. multocida *infections in neonates and young infants, a literature review was performed as follows: a PubMed database search covering 0-60 days of age with the search term “Pasteurella”, “neonate OR newborn OR baby”, “CNS infection OR brain abscess OR meningitis OR sepsis”. A total of 44 cases were reported in 38 papers between 1965 and 2022 [5,6,8-43], with neonates accounting for 31 (70.4%) of the cases. As routes of infection, traumatic exposure, including animal bites or scratches, was reported in six cases (13.6%), non-traumatic exposure, including licking or shared living space with animals, in 27 cases (61.3%), and vertical transmission from the mother in 11 cases (25.0%). Five patients (11.4%) died, and two patients (4.5%) were complicated by sequelae such as convulsions and paralysis. The patients requiring surgical intervention and those with neurological sequelae were all neonates (Table 2). 

**Table 2:** Clinical characteristics of 44 reported cases of P. multocida infection in neonates and young infants IQR: Interquartile range ^1^Vertical transmission was defined as documented evidence of *P. multocida* in organ(s) and/or body fluid in the mothers, and/or the patients developed pasteurellosis within 72 hours of age and without a history of neonatal exposure to animals. ^2^Duplication allowed

Characteristics (n=44)	Median (IQR) or n (%)
Age, days	21 (6.5–30)
Age distribution	
0–7 days	11 (25.0)
8–29 days	20 (45.4)
30–60 days	13 (29.5)
Animal exposure	
Traumatic	6 (13.6)
Non-traumatic (licked or shared living space with animals)	27 (61.3)
Mode of infection	
Horizontal	33 (75.0)
Vertical ^1^	11 (25.0)
Culture site ^2^	
Blood	39 (88.6)
Cerebrospinal fluid	39 (88.6)
Pharyngeal	5 (11.4)
Vagina	4 (9.1)
Unknown	1 (2.3)
Surgical procedure	
Drainage	4 (9.1)
Intravenous antibiotics (at any time) ^2^	
Ampicillin	24 (54.5)
Cefotaxime	22 (50.0)
Gentamicin	13 (29.5)
Meropenem, ampicillin/sulbactam, cefepime	2 (4.5)
Antibiotics, days	21 (14–21)
Outcome	
Survived	39 (88.6)
Without sequelae	35 (79.5)
With sequelae	2 (4.5)
Information on sequelae unknown	2 (4.5)
Died	5 (11.4)
Age (days) at the time of death	
0–7 days	5 (11.4)
8–29 days	0
30–60 days	0

Our patient experienced severe sequelae despite multidisciplinary management, including surgical drainage. Among the reported pediatric cases of *P. multocida* infection, the majority of cases with a poor prognosis were neonates or young infants [23,34,36,42,43]. Neonates generally have immature immune systems and are at high risk of serious bacterial infections [44]; therefore, special precautions should be taken for animal bites in newborns. 

*Pasteurella multocida* infection has several routes of transmission. In the present case, a minor dog bite on the cephalohematoma was considered to be the entry site. It is important for physicians to be aware that severe infections caused by* P. multocida* can occur even after minor or indirect contact with animals and not just from traumatic bites. Therefore, direct contact with pets should be avoided during the neonatal period. It is also important for family members to practice good hand hygiene when exposed to pet saliva.

In addition to antibiotic treatment, surgical procedures such as abscess drainage are sometimes required for severe *P. multocida* infections. In the literature review, 9.1% of cases required surgical intervention and all involved neonates. In the present case, antimicrobial therapy was promptly administered; however, the patient’s condition did not improve, and drainage was required on day five of hospitalization. Appropriate indications or timing of surgical intervention are unclear, and careful case-by-case determination of whether and when surgical intervention is necessary is required.

The need for prophylactic antimicrobials should also be considered. After the injury, the patient was monitored using only topical skin medications without systemic administration of antimicrobial agents. In some situations, such as moderate or severe bite wounds, puncture wounds, cat bite wounds, or wounds in immunocompromised hosts, the initiation of antimicrobial prophylaxis is recommended [45]. To the best of our knowledge, there are no specific guidelines for animal bites in neonates, and further investigations are required to determine when systemic antibiotics should be considered in neonates with immature immunity, as in our case.

## Conclusions

This case highlights the severe clinical course and poor prognosis associated with *P. multocida* infection in neonates, particularly following even minor trauma such as a dog bite. Given the high risk of severe outcomes, careful precautions should be taken when newborns come into contact with animals. Further research is necessary to establish clear guidelines on the management and prevention of* P. multocida *infections in neonates and infants younger than three months with immature immune systems.
